# SEPA+PrEP-BW intervention: A feasible and acceptable HIV prevention intervention for Black women

**DOI:** 10.1371/journal.pone.0336435

**Published:** 2025-11-18

**Authors:** Rosina Cianelli, Joseph P. De Santis, Evelyn Iriarte, Giovanna C. De Oliveira, Renessa Williams, Regine Placide Reaves, José G. Castro, Shanelle Hodge, Sophia O. Thomas, Carolyn Edwards, María José Baeza Robba, Natalia Villegas, Nilda Peragallo Montano

**Affiliations:** 1 School of Nursing and Health Studies, University of Miami, Coral Gables, Florida, United States of America; 2 Escuela de Enfermería, Pontificia Universidad Católica de Chile, Santiago, Chile; 3 College of Nursing, University of Colorado, Anschutz Medical Campus, United States of America; 4 Miller School of Medicine, University of Miami, Miami, Florida, United States of America; 5 Business and Management, University of Maryland Global Campus, Adelphi, Maryland, United States of America; 6 SEPA+PrEP-BW Community Advisory Board Member, United States of America; 7 SEPA+PrEP-BW Peer Facilitator, United States of America; 8 Center for Global Health Equity, University of Michigan, Ann Arbor, Michigan, United States of America; 9 School of Nursing, University of North Carolina at Chapel Hill, Chapel Hill, North Carolina, United States of America; Beth Israel Deaconess Medical Center/Harvard Medical School, UNITED STATES OF AMERICA

## Abstract

Although biomedical prevention strategies such as Pre-Exposure Prophylaxis (PrEP) are available, Black women (BW), who comprise less than 15% of the female population in the United States, account for 54% of new HIV infections among women. Many BW underestimate their HIV risk and face barriers to prevention, including traditional gender roles, racism, stigma, and medical mistrust. To address these challenges, we adapted SEPA+PrEP into SEPA+PrEP-BW, a novel biobehavioral HIV prevention intervention that integrates the empirically validated SEPA model (Salud/Health, Educación/Education, Prevención/Prevention, Autocuidado/Self-Care) with PrEP education and culturally relevant components tailored to BW. Using a mixed methods approach, we collected and analyzed quantitative and qualitative data from 73 BW residing in Miami-Dade County, Florida. Results demonstrated high feasibility (94.5% retention rate), strong acceptability (94.2% satisfaction with content), and unanimous willingness to recommend the intervention. Participants expressed interest in becoming peer facilitators and emphasized the importance of culturally relevant education and safe spaces for discussion. These findings support SEPA+PrEP-BW as a promising, community-driven intervention to address critical gaps in HIV prevention among BW.

## Introduction

Despite advancements in pre-exposure prophylaxis (PrEP) for HIV prevention, Black women (BW) are disproportionately affected by HIV; although they represent less than 15% of the female population, they account for 54% of new HIV infections among women in the United States (U.S.) [1]. The rate of HIV-related deaths among women in the U.S. is significantly higher for BW in comparison to non-Hispanic White women, representing a health disparity [2]. In Miami-Dade County (MDC), the study’s setting, HIV incidence is also disproportionately high among Black women; they are 10.3 times more likely than non-Hispanic White women to be diagnosed with HIV infection [3,4]. Further, because most BW (96%) in MDC who acquire HIV infection do so through heterosexual activity [4,5], MDC presents an ideal setting for planning and implementing interventions to reduce disparities in HIV prevention as well as related disparities (e.g., social determinants of health [SDoH]) among BW.

Ending the HIV Epidemic (EHE) is an initiative that aims to end the HIV epidemic in the United States by 2030 [6,7]. To achieve this goal, this initiative recommends that evidence-based interventions (EBI) should aim to increase HIV testing, PrEP awareness, access and maintenance, and consistent condom use, especially in populations disproportionately affected by HIV, such as BW [6–8]. The availability of PrEP for HIV prevention provides an unprecedented opportunity to slow and possibly stop the HIV epidemic. PrEP is a highly potent medication regimen that can be administered orally or intramuscularly and, when taken as prescribed, reduces the risk of HIV infection during sexual activity by 99% [9,10]. However, despite its effectiveness, only a small percentage (10%) of women who could benefit from PrEP were prescribed it in the U.S. in 2019 [11], and even fewer eligible individuals, particularly BW, were using PrEP. [9] Additionally, the National HIV Prevention Program Monitoring and Evaluation study (2021) found that only 35% of Black women diagnosed with HIV were aware of PrEP [12].

In comparison to non-Hispanic White females, BW had significantly lower rates of PrEP utilization in 2016 [13]. The limited awareness and use of PrEP among Black women are linked to various barriers to accessing HIV testing and PrEP services, including factors such as perceived racism, HIV stigma, and medical mistrust [14–16]. Moreover, BW often underestimate their risk of HIV infection and experience challenges in sexual negotiation related to traditional gender roles that hinder HIV prevention efforts [17,18]. Factors such as male dominance in sexual relationships, intimate partner violence, and insufficient sexual communication further hinder BW’s ability to engage in safer sexual behaviors [16,17]. While BW are tested for HIV during pregnancy, most do not have access to convenient testing locations, do not seek new testing unless they become pregnant again, and may not be aware of their HIV risk or status [19]. Moreover, experiences of racism and stigma contribute to increased anxiety and diminished self-efficacy, which in turn can lead to high-risk sexual behaviors such as engaging in unprotected sex with multiple partners [20–22]. Finally, BW frequently engage with community organizations to obtain services and information related to health, food distribution, childcare, employment opportunities, training, and other programs available to them [23]; thus, these community organizations provide an optimal setting for implementing HIV prevention interventions that are culturally tailored to the specific needs of the populations they serve.

Existing disparities highlight the urgency for HIV prevention interventions that serve BW and that incorporate PrEP [24]. To address this need, we adapted SEPA+PrEP into SEPA+PrEP-BW, a culturally tailored biobehavioral HIV prevention program that combines SEPA (Salud/Health, Educación/Education, Prevención/Prevention, Autocuidado/Self-Care) – an empirically validated behavioral HIV prevention intervention [25–27] – with education about the biomedical prevention strategy of PrEP [28]. This study aimed to investigate the feasibility and acceptability of the SEPA+PrEP-BW adapted intervention among BW.

## Methods

### Research design

We used a triangulation mixed methods approach to assess the feasibility and acceptability of SEPA+PrEP-BW among BW. This design involves collecting and analyzing quantitative and qualitative data concurrently to interpret the findings from different angles [29]. The current manuscript reports exclusively on Phase 3, in which we evaluated the feasibility and acceptability of the adapted SEPA+PrEP-BW intervention [30]. Phases 1 and 2 focused on identifying HIV prevention needs and adapting the intervention; we will report on those phases in a separate publication.

### Study setting

We conducted this study in southern Miami-Dade County (MDC), Florida, a community in which 22% of the population is Black [31]. Participants were recruited through different venues such as community organizations, public places, and online platforms (e.g., Facebook ads). The research team, along with members of the community-based organizations, conducted outreach to recruit potential participants at public locations that BW frequent, such as grocery stores, churches, and restaurants, and at locations where they work. We distributed flyers in predominantly Black neighborhoods, and encouraged Black community members to spread the word about the study. Recruitment began in December, 2022, and the final post-implementation focus group was conducted in May, 2024.

Our team has a 14-year history of successful collaboration in community health promotion activities and research projects, and our work is grounded in building mutually beneficial relationships with community partners to ensure that our efforts are impactful and responsive to the needs of those we serve. Three MDC community organizations worked with us in implementing the study. Our main partner, MUJER, introduced us to the two other community partners (Start Off Smart and Bridge to Hope) who participated in this study. We also created a Community Advisory Board (CAB).

### Participants

A convenience sample of 73 BW participated in this study. Eligibility criteria included a) self-identification as a Black woman (BW); b) aged 18–49; c) HIV antibody negative or unknown HIV serostatus; d) have engaged in sexual activity with a man in the last 6 months; e) have at least one sexual risk factor for HIV in the last six months (e.g., more than one sexual partner; unprotected sex; substance use before or during condomless sex with sexual partner[s]); f) speak and read English; and g) able to provide informed consent to participate. The [REDACTED] Institutional Review Board approved the study in June, 2022, and all participants provided verbal consent before completing the study assessments. Only the research team had access to the linking list that identified the participants. To help compensate for their time and transportation expenses, participants received an incentive of $50 per session to complete assessments and the 3 intervention sessions. BW who participated in the focus groups received an additional $50 incentive.

### SEPA+PrEP-BW intervention

SEPA+PrEP-BW is a culturally tailored, biobehavioral HIV prevention intervention designed to increase HIV testing, PrEP awareness and access, and consistent condom use among BW. The need for such an intervention is underscored by the CDC’s comprehensive list of effective HIV prevention programs, which includes only one intervention specifically directed to BW that incorporates PrEP [32]. This gap, along with community needs, national HIV prevention priorities, our CFAR grant support, and our prior experience with culturally grounded adaptations, informed the development of SEPA+PrEP-BW.

The intervention was adapted from SEPA+PrEP, an evidence-based program originally developed for Hispanic women [33], which itself builds on the SEPA intervention [25–27]. The adaptation followed the ADAPT-ITT model [33,34], preserving core theoretical foundations such as Bandura’s Social Cognitive Theory [35] and Freire’s Pedagogy [36], and incorporated input from a Community Advisory Board, healthcare providers, and community partners. A community-based participatory research (CBPR) approach [37,38] guided the process to ensure cultural relevance and responsiveness to the needs of BW.

### SEPA+PrEP-BW sessions to determine intervention feasibility and acceptability

We implemented 9 SEPA+PrEP-BW intervention groups with an average of 6–7 participants per group (range = 5–11). The intervention was delivered across 3 weekly 2-hour sessions by trained peer facilitators from the community. Each session included interactive discussions, role-playing exercises, and skill-building activities focused on HIV prevention, PrEP, and partner communication. Feasibility was assessed through attendance and retention rates, while acceptability was measured via participant feedback and focus group discussions [38,39].

### Data collection

Before joining the SEPA+PrEP-BW sessions, participants completed a socio-demographic questionnaire and responded to 5 questions about HIV risk and prevention. After each of the 3 SEPA+PrEP-BW sessions, participants completed a questionnaire composed of 4 open-ended questions about each session to assess satisfaction with the intervention. After the last session, participants also completed a questionnaire to assess the feasibility and acceptability of the intervention. Data were entered into secure web-based software (Qualtrics) to record their responses electronically.

Additionally, we invited 25 BW who completed the intervention to participate in one of 4 post-intervention focus groups. At the end of the third intervention session, participants were informed that they would receive an invitation to join a focus group via the email address they had provided. Invitations were sent to all 69 women who completed the intervention, and the first 25 who responded were included in one of the focus groups. The recorded focus group discussions and transcripts were stored in Box, a secure data storage platform.

### Study measures

We assessed socio-demographic information using a 17-item questionnaire to collect descriptive information about age, current relationship status, housing situation, religion, educational level, employment, income in U.S. dollars, and health insurance. Participants also responded to 4 questions about HIV risk and prevention: (1) *Have you been tested for HIV?* (yes, no); (2) *How concerned are you about getting HIV/AIDS?* (not at all, somewhat, extremely); (3) *How often did you use condoms during vaginal sex with your primary partner in the last three months*? (never, less than half of the time, more than half of the time, always, not applicable); and (4) *Have you heard about HIV Pre-Exposure Prophylaxis (PrEP?)* (yes, no).

We assessed feasibility – defined as the capacity of the intervention to be successfully implemented – through session attendance, retention rates, and completion of session feedback [38,39]. Our goal was to meet a threshold of at least 80% attendance at each of the 3 sessions. We assessed acceptability – defined as satisfaction with the intervention and the degree to which it met expectations – in two ways. At the end of each SEPA+PrEP session, participants responded to 4 open-ended questions: *What did you most like about this session*? *What did you least like about this session*? *Is there a topic you would like to repeat or clarify*? *Do you have other comments about the session?* At the end of session 3, participants completed a 14-item acceptability questionnaire [38,39]. This measure included 11 multiple-selection and 3 open-ended questions assessing satisfaction with SEPA+PrEP-BW’s content and activities, clarity and comfort, the relationship between facilitators and participants, and expectations about the intervention. The questionnaire also elicited suggestions about how to improve the intervention [38].

Twenty-five BW who completed all 3 SEPA+PrEP-BW sessions participated in one of four 45–60-minute focus groups. These focus groups allowed us to gather qualitative data, offering deeper insights into the participants’ intervention experiences and providing context for the quantitative data collected. The focus groups were conducted via Zoom® and audio-recorded for accuracy (with participants’ permission). To guide the focus groups, we developed a semi-structured interview guide comprised of open-ended questions drawn from existing literature and from our experience implementing HIV prevention programs. Sample questions included: a) *What do you think about SEPA+PrEP-BW?* b) *Do you think SEPA+PrEP-BW responds to your community’s needs?* and c) *Do you think SEPA+PrEP-BW should be implemented with other groups of people?* Audio recordings were transcribed verbatim and securely stored in a locked office, with digital files saved on password-protected computers to maintain participants’ confidentiality and data integrity.

### Data analysis

We conducted quantitative analyses using the Statistical Package for Social Sciences (SPSS) version 28.0, employing descriptive statistics and frequencies. We analyzed focus group data through conventional content analysis to identify qualitative content that triangulated participants’ quantitative responses [29,40]. While these two data types are analyzed separately, their results are integrated and interpreted together. This integrated, or triangulated, approach allowed us to identify how the quantitative and qualitative data complement and support each other in providing insights into our research questions [29,40].

## Results

### Socio-demographic characteristics

A total of 73 BW, with a mean age of 36 years (*SD* = 8.5; range 18–49), completed the socio-demographic questionnaire (Table 1). All participants resided in MDC, Florida and had completed a mean of 11.8 years of education (*SD* = 1.7; range 4–16). Most were either single (57.5%; *n* = 42) or in a relationship, but not legally married (27.4%; *n* = 20). Almost two-thirds (64.4%; *n* = 47) were unemployed, and 72.6% (*n* = 53) reported having Medicaid as health insurance.

**Table 1:** SEPA+PrEP-BW participants’ socio-demographic characteristics (*N* = 73).

Variables	*n* (%) or *M* (*SD*; range)
**Age (in years)**	36 (*8.5*; 18-49)
**Ethnicity**	
African American	64 (87.7)
Hispanic	5 (6.8)
Haitian American	3 (4.1)
Native American	1 (1.4)
**Relationship status**	
Single	42 (57.5)
In a relationship, not legally married	20 (27.4)
Married	8 (11)
Separated	3 (4.1)
**Years of education**	11.8 (*1.7*; 4-16)
Primary education, no diploma	20 (27.4)
High school graduate	40 (54.8)
College	13 (17.8)
**Employment status**	
Unemployed	47 (64.4)
Employed	26 (35.6)
**Monthly household income**	
$0-$499	30 (41.1)
$500-$999	16 (21.9)
$1000-$1999	13 (17.8)
$2000-$2999	7 (9.6)
$3000-$3999	5 (6.8)
$4000+	2 (2.7)
**Health insurance** Medicaid	53 (72.6)
Private insurance	6 (8.2)
Other (out of pocket, don’t pay)	14 (19.2)

*Note. M* = mean; *n* = number; *SD* = standard deviation.

### HIV risk and prevention

Almost all of the participants (91.8%; *n* = 67) reported having been tested for HIV in their lifetime, while 8.2% (*n* = 6) reported never having been tested. When asked how often they had used condoms during vaginal sex in the last 3 months, 80.8% (*n* = 59) reported never using a condom or using it inconsistently while only 19.2% (*n* = 14) reported always having used a condom; notably, during the SEPA+PrEP-BW sessions, participants revealed that their partners were unwilling to use condoms. In addition, almost three-quarters (73.9%; *n* = 54) of the participants reported that they were not concerned or only somewhat concerned about acquiring HIV (Table 2). Finally, while 53.4% (*n* = 39) of participants indicated that they were aware of PrEP before the intervention, during the SEPA+PrEP-BW sessions it became evident that a significant number of the BW had confused PrEP with antiretroviral therapy for HIV; further, there was a prevalent misconception among participants that antiretroviral therapy and PrEP can be used only by men who have sex with men.

**Table 2:** SEPA+PrEP-BW HIV prevention behaviors before participation (N = 73).

Questions	*n* (%)
**How worried are you about getting HIV/AIDS?**	
Not at all	32 (43.8)
Somewhat	22 (30.1)
Extremely	19 (26.1)
**Do you know about HIV PrEP?**	
Yes	39 (53.4)
No	34 (46.6)
**How often do you use a condom during vaginal sex?**	
Never	34 (46.6)
About half the time	12 (16.4%)
More than half the time	13 (17.8%)
Always	14 (19.2)

*Note. M* = mean; *n* = number; *SD* = standard deviation.

### Intervention feasibility and acceptability

Of the 73 participants, 69 completed all 3 sessions of the SEPA+PrEP-BW intervention. One participant completed the demographic assessment but did not attend the first session, while 3 completed the first session only (of these, one could not finish the intervention due to relocation to another state, and 2 could not be contacted following their initial session). Additionally, 25 of the BW participated in the post-intervention focus groups (Table 3).

**Table 3:** Acceptability of the SEPA+PrEP-BW intervention (*n* = 69).

Questions	*n* (%) or *M* (*SD*; range)
**Satisfaction with intervention content and activities**	
Rate your satisfaction with the information	4.8 (*0.8*; 1-5)
Satisfied/Very satisfied	65 (94.2)
Neutral	2 (2.9)
Dissatisfied/Very dissatisfied	2 (2.9)
Rate your satisfaction with the activities	4.8 (*0.7*; 1-5)
Satisfied/Very satisfied	67 (97.1)
Neutral	0 (0)
Dissatisfied/Very dissatisfied	2 (2.9)
**Clarity & comfort**	
Rate your understanding of the information delivered	4.3 (*1.3*; 1-5)
High/Very high	56 (81.2)
Neutral	6 (8.7)
Low/Very low	7 (10.1)
Rate your level of comfort with the examples/pictures used	4.4 (*1.1*; 1-5)
High/Very high	59 (85.5)
Neutral	6 (8.7)
Low/Very low	4 (5.7)
Did the sessions include topics/issues that you confront?	
Yes	48 (69.6)
Partially	18 (26.1)
No	3 (4.3)
**Relationship between facilitators and participants**	
Rate your satisfaction with the facilitators	4.7 (*0.9*; 1-5)
Satisfied/Very satisfied	62 (89.8)
Neutral	5 (7.2)
Dissatisfied/Very dissatisfied	2 (2.9)
**Expectations**	
Did the sessions meet your initial expectations?	
Yes	61 (88.4)
Partially	4 (5.8)
No	4 (5.8)
How did you feel participating in the sessions?	4.8 (*0.7*; 1-5)
Satisfied/Very satisfied	64 (92.7)
Neutral	4 (5.8)
Dissatisfied/Very dissatisfied	1 (1.4)
Would you participate in other programs like SEPA+PrEP-BW?	
Yes	66 (95.7)
No	3 (4.3)
Would you recommend SEPA+PrEP-BW?	
Yes	69 (100)
No	0 (0)

*Note. M* = mean; *n* = number; *SD* = standard deviation.

### Satisfaction with the intervention content and activities

We asked participants to rate their satisfaction with the intervention content and scored their responses on a 5-point scale (1 = *very dissatisfied*; 5 = *very satisfied*). Almost all of the participants were “satisfied/very satisfied” (94.2%, *n* = 65) with the information provided, and “satisfied/very satisfied” 97.1% (*n* = 67) with the intervention activities. In the focus groups, one participant noted:

*I thought it was informative. I thought it was necessary. I appreciated the fact that it was in a group format, so you could hear other people’s thoughts and hear their opinions. I liked that we learned from people directly in our community as well as the shared information. So, I thought it was a very effective way of providing the information to our community.*

Only 2 participants indicated that they were “dissatisfied/very dissatisfied” with the intervention content or activities; however, this feedback was not clearly captured during the focus groups. One participant stated: *“I don’t want to do any writing. I do too much writing at school. I do not want to do any homework…like the evaluations we did at the beginning.”*

### Clarity and comfort: Understanding of the information delivered

We asked participants to rate their understanding of the information delivered during the SEPA+PrEP-BW sessions and scored their responses on a 5-point scale (1 = *very low*; 5 = *very high*). Most participants (81.2%, *n* = 56) reported a “high/very high” understanding of the content, while 8.7% (*n* = 6) reported “neutrally understanding” and 10.1% (*n* = 7) reported “low/very low” understanding (*M* = 4.3; *SD* = 1.3; range = 1–5). Focus group participants reported that the sessions provided useful information that the women could share with their families and with members of their community. However, comments indicating neutral or low levels of understanding were not captured during the focus group discussions. One participant remarked:

*I felt like I could really use the information. I think it’s so important because when I’m telling somebody about something like PrEP, I hope that they tell their friends to tell their friends, to tell their friends, so that this information can spread.*

### Methodologies used to enhance learning

We asked participants to rate their comfort level with the images, movies, and examples used during the SEPA+PrEP-BW sessions and scored their responses on a 5-point scale (1 = *very low comfort*; 5 = *very high comfort*). Most participants (85.5%; *n* = 59) reported a high or very high level of comfort with the content, while 8.7% (*n* = 6) reported a “neutral” comfort level and 5.7% (*n* = 4) reported a “low/very low” comfort level (*M* = 4.4.; *SD* = 1.1; range = 1–5). One participant expressed: “*I learned a lot. We got a lot of information. I learned a lot of things that I didn’t know.*” Yet another participant emphasized the importance of showing pictures related to STDs from a preventive point of view; she verbalized: *“I bet [if] you showed the pictures to them [kids] they will not do a lot of stuff.”*

### Relationship between facilitators and participants: Satisfaction with the peer facilitators

We asked participants to rate their satisfaction with the peer facilitators and scored their responses on a 5-point scale (1 = *very low*; 5 = *very high*). Almost all (89.8%; *n* = 62) reported high or very high satisfaction with the peer facilitators (*M* = 4.6; *SD* = 0.9; range = 1–5). In addition, many participants were highly interested in becoming facilitators for similar studies to be conducted in the future. One participant stated:

*Oh, wow! I would definitely be interested in doing something like that. I would love to be a part of something like that, because I feel like it’ll give me, you know, just purpose other than just having, like, you know, a job where I’m making money. I think doing something that could actually make a difference for somebody like that definitely be something that I would, you know, love to be a part of.*

### Experiences in the intervention and perceived advantages

All focus group participants reported that the sessions were immensely informative and easy to understand. Most participants stated that they prefer in-person meetings, as opposed to attending sessions via Zoom®. Many participants expressed that Zoom® would be less engaging and less confidential; others stated that their children at home may be distracting.

Several women reported leaving the sessions feeling empowered and connected to each other. One participant commented: *“The sessions were incredibly effective and successful. I think it made people comfortable, and I want to talk so… I thank you all so much.”* Another added, “*I think it [the intervention] was just incredibly effective. I think it was really successful.”* Yet another participant expressed:

*It’s like, whatever problems that you have, you can actually express them [during the sessions]. You could get an answer instead of like in a big group. [In a big group] everybody’s asking questions. Your questions are never gonna get asked.*

Finally, additional participants expressed enjoying the openness and transparency of the discussions and stated that they were happy to share their experiences without feeling judged.

### Implementation in the community

Participants suggested that the SEPA+PrEP-BW intervention should also be offered to men and adolescents. Due to a lack of education and available information in the community, many participants affirmed that sessions should be implemented in local middle schools and high schools. One participant shared:

*I would suggest that in order for you guys to reach the target audience of where you know where this issue is predominant, I think that it is important, like the other young lady mentioned, to educate young girls while they’re in school, like go to, you know, high schools.*

Many of the women recognized that efforts should be made to help people feel more comfortable talking about HIV in the community. They proposed using various social media platforms, such as Facebook, Instagram, TikTok, and WhatsApp, to advertise and promote the SEPA+PrEP-BW intervention.

### Potential adaptations

All participants stated that there are currently no similar programs in the community to educate residents on HIV prevention. Many requested more weekly sessions, with one commenting: *“I wanted a little more time. It was very interesting.”* Several women suggested including more hands-on activities and detailed role-play scenarios. Participants also agreed that offering additional sessions for BW at different locations in MDC neighborhoods would be beneficial. Lastly, participants requested more information and discussion about peer pressure, teen pregnancy, mental health issues, and intimate partner violence. All participants who completed all 3 sessions (*n* = 69) also provided feedback at the end of each session. The retention rate was 94.5% (N = 73).

## Discussion

This study evaluated the feasibility and acceptability of the SEPA+PrEP-BW intervention among BW in Miami-Dade County (MDC), a population disproportionately affected by HIV. The findings demonstrate that the intervention was well-received, with high levels of participant satisfaction, high engagement, and a retention rate of 94.5%. Remarkably, 69 of the 73 participants attended every session, indicating strong commitment to and involvement in the intervention. Further, the completion of session feedback by all participants reflects their active investment in enhancing the intervention for the benefit of others in the community.

These results suggest that SEPA+PrEP-BW is both feasible to implement and acceptable within this community setting. Further, the results highlight the HIV risks faced by BW and emphasize the need for effective, culturally tailored biobehavioral prevention interventions such as SEPA+PrEP-BW that incorporate HIV testing, PrEP, and condom use.

The intervention’s success is consistent with prior research showing that culturally tailored HIV prevention programs are more effective in reaching minority populations, including BW [41,42]. Participants reported high satisfaction with the content and delivery of the sessions, and many expressed interest in becoming peer facilitators—an indicator of community ownership and intervention sustainability. They also noted that the SEPA+PrEP-BW intervention included relevant cultural content, engaging activities, and diverse features, underscoring its feasibility for BW. These findings are significant as they suggest that BW participants felt comfortable with the intervention’s format, activities, and facilitators.

Our findings align with studies emphasizing the importance of community-based participatory approaches and peer-led models in HIV prevention [37,38,43]. The involvement of the community partners ensures that interventions are tailored to reflect the perspectives and experiences of the community served, ultimately enhancing the relevance and effectiveness of prevention interventions.

Importantly, SEPA+PrEP-BW addressed key barriers to HIV prevention identified in the literature, such as low awareness of PrEP, inconsistent condom use, and limited access to culturally relevant education [14,15]. Participants’ feedback highlighted the value of interactive learning, peer facilitators, and safe spaces for discussion. These elements contributed to the intervention’s acceptability, suggesting that SEPA+PrEP-BW could be scaled up across demographically similar communities at high risk for HIV. The intervention’s feasibility was further supported by strong attendance and retention, as well as participants’ willingness to recommend the program and engage in future sessions. The use of a CBPR framework and collaboration with trusted community organizations helped foster trust and relevance, which are critical for successful implementation in historically marginalized communities [38,43].

These findings align with broader public health goals, including the CDC’s “Ending the HIV Epidemic” initiative, which emphasizes the need for scalable, evidence-based interventions that increase PrEP awareness and uptake and reduce HIV transmission in high-risk populations [7]. SEPA+PrEP-BW’s integration of behavioral and biomedical strategies positions it as a strong candidate for broader dissemination. Future research will focus on evaluating the efficacy of the SEPA+PrEP-BW intervention and its potential for broader implementation across diverse settings (e.g., clinics, faith-based organizations, and primary care settings).

### Strengths and Limitations

Strengths of the study include its mixed-methods design, high participant retention, and strong community engagement. The integration of qualitative and quantitative data provided a comprehensive understanding of the participants’ experiences and the impact of the intervention. Limitations must be considered in light of the study findings. First, the self-report nature of data collected may have influenced outcomes. Second, the cross-sectional nature of this study does not permit longitudinal examinations of the intervention over time. Third, the use of convenience sampling restricts the findings’ generalizability. These results should not be considered representative of all BW in the U.S.

## Conclusion

This study successfully demonstrates the feasibility and acceptability of SEPA+PrEP-BW, an HIV biobehavioral intervention culturally tailored to BW that addresses critical gaps in HIV prevention strategies. Participants’ strong engagement and positive feedback underscore the intervention’s relevance and the importance of the CBPR model in the design and implementation of health initiatives. Our next step will be to test the efficacy of the SEPA+PrEP-BW intervention and to explore pre-implementation strategies to ensure its sustainability and broader impact in reducing HIV risk among BW.

## Supporting information

S1 File Demographic. (XLSX)

S2 File Feasibility & Acceptability. (XLSX)
